# Supplementary material for: Using genetic data to identify transmission risk factors: Statistical assessment and application to tuberculosis transmission
Source: PLoS Comput Biol. 2022 Dec 5;18(12):e1010696. doi: 10.1371/journal.pcbi.1010696 (PMC9754595; doi:10.1371/journal.pcbi.1010696)
Supplement: Attachment — Submitted filename: Letter_Response.pdf [file pcbi.1010696.s004.pdf]

September 20, 2022

Dr. Nicola Segata and Dr. Thomas Leitner
Associate Editor
PLoS Computational Biology

Dear Drs. Segata and Leitner,

My co-authors and I have reviewed and approved the revision of our manuscript entitled "Using genetic data to identify transmission risk factors: statistical assessment and application to tuberculosis transmission." We want to thank you and the referees for the constructive feedback which helped us to improve our work. We followed all the comments carefully and addressed the remaining issues in our paper. In what follows, we provide a point-by-point response to their comments and relevant changes in the manuscript.

Best regards,

Sanghyuk Shin
Associate Professor
University of California, Irvine

Response to the reviewers' comments on
Using genetic data to identify transmission risk factors: statistical assessment and application to tuberculosis transmission

Comments from Reviewer 1

TransPhylo output assigns to each case a probability to be an infector/infecctee. This probability can be used to establish a threshold on how much you can rely in the prediction. This is important to avoid spurious assignment of infector/infectee status. However, it is not clear to me when and how this parameter has been used in the in the real dataset (there is a mention to it for the simulated dataset) and if so, which is the impact of changing the threshold in a real-world scenario?

> ***Reply***:
> *We agree it is currently not clear what the cutoff used for the real data analysis is. It is the same cutoff as was used in the simulations. We have added a sentence to the methods section to make this clear. In practice, we have found the distribution of probabilities of being an infection source to be extremely bi-modal. What this means is that changing the threshold probability used to decide who is and is not an infection source does not dramatically impact the results of our analysis. We can see this by changing the threshold and counting how many labels are switched when we change the threshold. In the real data analysis 5 SNP data set, a threshold of 0.4 instead of 0.6 results in a single additional infection source, a threshold of 0.8 results in 5 fewer infection sources. In the 10 SNP data set, a threshold of 0.4 results in 17 (4% of study participants) new infection sources, a threshold of 0.8 results in 9 (2.4% of study participants) fewer infection sources. We are confident these changes are small enough for the exact threshold to not matter. We have added this information to the appendix.*

Relevant changes in the manuscript:

Methods

This same cutoff probability of 0.6 was used for the real data analysis as well.

While we cannot assess sensitivity and specificity of `Transphylo` on real data, we can at least look at the distribution of probabilities of being an infection source to see how robust our results are to changes in the threshold probability used to assign labels. The distribution of probabilities of being an infection source was likewise bi-modal in the real data analysis. In the 5 SNP data set, a threshold of 0.4 instead of 0.6 resulted in a single additional infection source, a threshold of 0.8 resulted in 5 fewer infection sources. In the 10 SNP data set, a threshold of 0.4 results in 17 (4% of study participants) new infection sources, a threshold of 0.8 results in 9 (2.4% of study participants) fewer infection sources.

The authors fail to find a negative association between HIV status and transmission. This is really interesting and very much in the line of other works using different approaches. But as the authors mention there are also reports for the contrary , and many times it is assumed PLHIV tend to transmit less. The failure to find an association maybe a real signal or just a limitation of the TransPhylo approach in the Botswana dataset. To rule out the second possibility I wonder if there is a problem on how an infectee is defined. For infectors you define those with > 0.6 probability while for infectees you will expect to use only those with < 0.4. Is that the case? Otherwise you will pool in the same infectees pot cases with a clear signal to be infectees with cases with no signal either in one direction or the other, this will dilute size of the studied effect.

Reply:
Infection source status is defined using a cutoff probability of 0.6, we have made this clearer in the methods section (see previous comment). An example of how this plays out in practice: Suppose across one thousand posterior samples, individual A infects individual B in thirty percent of the samples, individual C in twenty percent of the samples, and individual D in thirty percent of the samples. It is true that we would conclude there is a low probability that A infected any of these individuals in particular. However, the overall probability that A is an infection source, of having infected at least one other person, would be 0.8. In this scenario, A would be decisively labeled an infection source using our chosen threshold probability of 0.6. Thus, in a world where infection source probabilities are accurately calculated, we are not worried about possibly diluting the size of the studied effect based on how labels are assigned, especially because in practice these probabilities tend to be clustered around 0 or 1 (see previous comment). We agree the failure to find an association may be a real signal or a result of using `TransPhylo` in a low density sampling setting. Unfortunately we do not think this question can be easily evaluated with the present data set, as the true infection source labels cannot be observed, so we will never know the accuracy of the infection source probabilities we calculated.

Ascertainment bias and substitution rates. Please clarify if the analysis has been done only with SNP alignment or have been corrected for ascertainment bias (by the number

of invariant sites). The second analysis is the correct one as it allows to use the known molecular clock rates calculated for Mtb.

Reply:
We analyzed only varying sites, and used an ascertainment bias correction to account for this. The relevant passage starts on line 309 of the previously submitted text and reads "We used an HKY model with strict molecular clock and a constant effective population size model, and used an ascertainment bias correction for sequences of unknown length to account for the fact that analyzed sequences included only varying sites(1)."

Relevant changes in the manuscript: None at this time.

In addition, please clarify which molecular clock rate has been used and which is the rationale to use it (also because you have different lineages, do you think in using different rates for different lineages?)

Reply:
We used a uniform prior on the clock rate ranging from 1 x 10-8 to 5 x 10-7, which includes the estimated clock rates across lineages reported by Menardo et al(2). We believe this should account for the fact we have samples from multiple lineages. The clock rate was scaled by the number of varying sites for each cluster to correct for the fact that we used BEAST2 on SNPs, rather than the full genome. This information was included in the Supplementary File in the initial submission, but has now been moved to the main text and revised for clarity.

Relevant changes in the manuscript:

Methods

We used a uniform prior for the molecular clock with a lower bound 1×10^{-8} and upper bound 5×10^{-7} scaled by 4.2 million divided by the number of sites in the data for each cluster. The scaling was done to account for the fact that we used BEAST2 on SNPs, not the full genome. The upper and lower bounds were chosen based on work by Menardo et. al, and were chosen to be suitable for all TB lineages (2). Priors on the frequency parameter, kappa and population size were all log normal distributions with parameter M set to 1.0 and parameters S set to 1.25.

Please, following the FAIR principles, deposit all the relevant data in repositories and provide accession (for sequence data) and DOI numbers for the metadata. Analysis must be reproducible and therefore please include the minimum information (sampling date, HIV status or any other variable needed to reproduce the analysis)

Reply:
All code and data needed to reproduce this paper are available at `https://github.com/igoldsteinh/kopanyo_tp_code` *— a github repository containing SNP alignments, meta data, BEAST XML model specification files, and R code reproducing resutls of the paper. We have updated the manuscript to include a link to the github repository.*

Relevant changes in the manuscript:

Methods

All code and data needed to recreate the results of this study are available at `https://github.com/igoldsteinh/kopanyo_tp_code`**.**

It is not clear to me which the final % of cases sequenced out of the total expected in the population?

Reply:
We calculated the percent of TB cases in Botswana for which we had sequences as compared to the total expected number of TB cases during this time period. We have added additional details to the methods section explaining how we calculated this quantity.

Relevant changes in the manuscript:

Methods

For example, due to under-diagnosis, the World Health Organization estimated that only 54% of people with new TB episodes in Botswana were reported in 2019(3). Using this proportion, we divided the number of M. tuberculosis sequences available by the number of estimated TB episodes (reported + unreported) to calculate the sampling proportion for our study. In more detail: we divided the number of diagnosed incidents of TB during the Kopanyo study (N = 4,331) by 0.538 to determine the total number of reported and unreported TB episodes (N = 8049). We then divided the number of sequences initially available for analysis (N = 1306) by 8049 to determine the sampling proportion of 16%. We continued to use this conservative sampling proportion estimate after increasing our sequenced samples to 1426.

In general, given the extensive simulation work, can the authors suggests a coverage threshold from which TransPhylo can be applied with more confidence? This will help guide/design studies aiming to incorporate TransPhylo.

Reply:
We feel the answer to the question of what sampling proportion is needed in order to answer scientific questions using `TransPhylo` *is likely to be disease and question specific. As such there is no one recommendation we can make which would cover every situation where* `TransPhylo` *might be used. Just as it is typical to conduct a power analysis for pre-specified effect sizes in clinical trials, we would likewise recommend conducting a simulation study with pre-specified effect sizes to determine an appropriate sampling proportion before conducting a real world study using* `TransPhylo`. *Interested researchers could use our code as a starting point for conducting such a simulation study. In fact, members of our team have already done this as part of a grant application to study transmission risk factors for SARS-Cov-2. We have added a paragraph to the Discussion discussing this option.*

Relevant changes in the manuscript:

Discussion

Simulation studies with pre-specified effect sizes could be used as part of study design in order to help researchers decide what sampling protocol they should aim for in future studies. The code used in this study could be used as a starting point for conducting such simulation studies.

Comments from Reviewer 2

Line 37: This sentence is confusing. It would probably be better to simply remove everything after "when the response…"

Reply:
The revised sentence is quoted in full below.

Relevant changes in the manuscript:

Introduction

We evaluate bias and precision of the estimators of the odds ratios for probability of being an infection source.

Lines 43-44: It isn't clear what the "response variable" means in this context making it difficult to understand this sentence.

Reply:
The revised sentence is quoted in full below.

Relevant changes in the manuscript:

Introduction

In particular, we evaluate the properties of using `TransPhylo` generated infection source labels and logistic regression, and `TransPhylo` infection source labels with two different statistical models that take into account measurement error.

Lines 51-57: This is a discussion of the results and should not be in the introduction.

Reply:
The passage has been removed from the text.

Relevant changes in the manuscript:

Introduction

DELETED: Our results show that performance of these pipelines varies dramatically depending on the true odds ratio. For modest effect sizes, tests using these estimators fail to reject that the odds ratio is one regularly. Measurement error models often increase bias and confidence interval width past the point of usefulness. However, increasing proportion of cases sampled, duration of sampling period, or total sample size may improve pipeline performance. Overall, our findings suggest these pipelines should be used with caution, as their performance will depend highly on situational context.

Lines 69-73: This sentence is hard to understand because of the use of the term "disease recipient". The word "recipient" makes the term sounds like it is referring to the infectee in a given infector-infectee link. Therefore, a "disease recipient" can't also be an "infection source" in a given infection event. However, it seems like the term is being used to simply mean an individual in an outbreak, i.e. a case. This is a general issue throughout the methods that makes it hard to track. I think instead of using "disease recipient" or "infection recipient" to refer to an individual in the simulation it would be better to use a generic word like simply "individual" or "case". This would greatly aid in clarifying the methods.

Reply:
We have replaced the words "disease recipient" and "infection recipient" throughout the manuscript with the words "individual" and "case", whichever is most clear in our opinion. We will refrain from including all sections of the text with changed words, but include the original sentence highlighted by the reviewer below as an example. We hope this change has made this sentence more clear.

Relevant changes in the manuscript:

Methods

Using the posterior distribution of transmission trees, we can create a probability of being an infectious source for each sampled individual by calculating the proportion of posterior trees in which the individual transmitted the disease to another individual (regardless of whether the individual was sampled).

Lines 104-106: It is unclear why the authors use 3 times lower and then 3.33 times higher? Why not 3 times higher? But, in the rest of the paper it seems like they did use 3 times higher, so then why does it say 3.33 times here?

Reply:
We have rephrased for clarity and to match the notation used in the rest of the manuscript. The choice to use 0.3 instead of 0.33 was arbitrary (3.33 times higher vs 3 times higher using the old language in the manuscript). We wanted a simulation setting where the ratio of interest was quite small, what we would subjectively think of as a large effect size. We feel the current settings accomplish this goal adequately.

Relevant changes in the manuscript:

Methods

The settings were: probability of transmission given contact was 3.02 times higher among people living without HIV compared to that among people with HIV, 1.75 times higher, equal, 1.75 times lower, or 3.33 times lower. All other parameters were identical for the two classes of cases. For the 3.02 setting, we will round down and refer to this as the 3 setting for the duration of the paper.

Line 108 and 112: It is not clear from earlier in this section what a set of clusters would be. The description of the simulation set up seems to imply that 1 simulation = 1 outbreak = 1 cluster. What then would differentiate 100 sets of 50 clusters from 5000 simulations (in other words 5000 clusters)? This should be clarified. Similarly, the sentence "only simulations where all fifty clusters had at least two sampled individuals were accepted" is not clear in light of the previous simulation description.

Reply:
We have clarified the language describing simulations (see below). It is true there is little difference between 100 sets of 50 clusters, and 5000 separate clusters. We chose this method of simulation so that we could use the multiple cluster functionality of `TransPhylo` which allows us to analyze multiple phylogenetic trees simultaneously. We tested this functionality as it is also the functionality used in the real data analysis.

Relevant changes in the manuscript:

Methods

A single simulation consisted of fifty simulated clusters, where each cluster lasted eight years. From a single cluster, individuals were sampled from the last three years of the outbreak. Recipients were only sampled during their infectious periods, with the sampling time equally likely at any point in this time frame. For each cluster, we sampled 16% of eligible recipients for each of the last three years of the outbreak. Only simulations where all fifty clusters had at least two sampled individuals were accepted. In addition, because individuals could be active in multiple years, there were cases where there were not enough individuals in a particular year left to reach the percentage of 16%, simulations were likewise rejected in this scenario.
For each simulation setting, we generated one hundred simulations.
We used the multiple cluster functionality of `TransPhylo` to analyze all simulated trees from a single simulation simultaneously.

Lines 116-121: Since there was a clear method for getting the value of 16% it would be helpful to provide the calculation of how to go from 54% to 16% including the values used for the number of "sequences available" and "number of estimated TB episodes" with sources.

Reply:
We have added additional details to the methods section with the requested details.

Relevant changes in the manuscript:

Methods

In more detail: we divided the number of diagnosed incidents of TB during the Kopanyo study (N = 4,331) by 53.8% to determine the total number of reported and unreported TB episodes (N = 8049). We then divided the number of sequences initially available for analysis (N = 1306) by 8049 to determine the sampling proportion of 16%. We continued to use this conservative sampling proportion estimate after increasing our sequenced samples to 1426.

Line 123: New sentence should start after "nosi"

Reply:
See below for the revised sentences.

Relevant changes in the manuscript:

Methods

Once sampled, we generated timed phylogenetic trees for each cluster using `nosoi`. The trees from separate clusters represent the trees from separate transmission clusters which would be generated as part of a real data analysis.

Lines 136-138: Remove "in the second setting" and "in the third setting", they only make the paragraph more confusing. It would also be very helpful to have a supplemental table that lists all of the different simulation scenarios split by primary and secondary including the effect size, number of clusters, sampling proportion, sampling window, and anything other inputs that changed between the settings.

> ***Reply***:
> *See below for the revised sentences. We agree a table summarising the simulation settings would be helpful and have added one to the appendix.*

Relevant changes in the manuscript:

Methods

To investigate the impact of increasing the sampling density, we increased the sampling proportion from 16% to 32% while keeping the sample size approximately the same by halving the number of clusters in a simulation (from fifty to twenty-five). To further investigate the consequences of increasing sampling density, we then increased the sampling proportion from 16% to 64%, again keeping the sample size approximately similar using only 13 clusters. To investigate the impact of an increase in sample size, we kept the sampling proportion at 16%, but doubled the number of clusters from fifty to one hundred. To investigate the impact of shortening the time from beginning of epidemic to beginning of sampling, we kept the sampling proportion at 16%, but increased the sampling window from the last three years to the last seven years of the simulation, while decreasing the number of clusters from fifty to thirty-five to approximately maintain the original sample size. All simulation settings are summarised in Appendix Table 1

Appendix

Setting	True OR	Clusters per Sim.	Samp. Prop.	Samp. Window
0.3	0.25	50	0.16	3
0.57	0.52	50	0.16	3
1	1	50	0.16	3
1.75	1.94	50	0.16	3
3	3.53	50	0.16	3
Increase Sample Size	1.94	100	0.16	3
Double Sampling Density	1.94	25	0.32	3
Quad Sampling Density	1.94	13	0.64	3
Increase Sampling Window	1.94	35	0.16	7

Table 1: Simulation settings used in this study.

Line 144: What are "all disease parameters"? The ones described below?

> ***Reply***:
> *All disease parameters referred to all parameters in* `TransPhylo` *which govern infectious disease dynamics. In practice, this is all parameters which are not the sampling proportion. We have changed the language to make this less confusing, see below.*

Relevant changes in the manuscript:

Methods

The sampling proportion π was allowed to vary among clusters, while all other parameters were shared across all trees.

Line 147: Does "sampling prior" mean the sampling proportion prior? This is another place where a supplemental table listing all of the parameters and their prior distributions would be helpful.

> ***Reply***:
> *It is the prior for the sampling proportion. We have clarified the language, and added a supplemental table with all parameters and prior distributions, see below.*

Relevant changes in the manuscript:

Methods

Based on empirical results from early simulations, for the primary simulations, the prior for the sampling proportion was set as a beta distribution with alpha equal to 1 and beta equal to 19. Parameters and their priors are summarised in Appendix Table 2.

Appendix

Parameter	Defn.	Prior	Value/Prior Median & 95% Quantiles
π	Sampling prop.	Beta(1,19)	0.036 (0.0014, 0.18)
r	Reproduction number	Exp(1)	0.68 (0.023, 3.70)
p	No effect	NA	0.5
N_{eg}	W/in-host ESS	Exp(1)	0.68 (0.023, 3.70)
α_{gen}	Gen. time shape	NA	10
β_{gen}	Gen. time scale	NA	0.1
α_{samp}	Samp. time shape	NA	10
β_{samp}	Samp. time scale	NA	0.1

Table 2: `TransPhylo` parameters and priors

Line 165: It is reverse than you would expect for HIV to be 1 when the individual is HIV- and 0 if the individual is HIV+. It doesn't make a difference but using this coding, it would be clearer to have "HIV-" in the equation instead of just "HIV".

Reply:
We agree this could be clearer, and we have changed to HIV^- in the two places this appears in the methods section, see below

Relevant changes in the manuscript:

Methods

$$\log\left(\frac{P(Y=1)}{1-P(Y=1)}\right) = \beta_0 + HIV^{-}\beta_1,$$

$$\begin{aligned}
\beta_0 &\sim N(0,1),\\
\beta_1 &\sim N(0,1),\\
\log\frac{P(Z_i=1)}{1-P(Z_i=1)} &= \beta_0 + HIV_i^{-}\beta_1,\\
Y_i \mid Z_i = 0 &\sim \textbf{Bernoulli}(p = 1 - \textbf{Specificity}),\\
Y_i \mid Z_i = 1 &\sim \textbf{Bernoulli}(p = \textbf{Sensitivity}).
\end{aligned}$$

Table 1: It says this is for 100 simulations. Is that 100 clusters? 100 sets of 50 clusters so 5000 clusters? This should be clarified. Also, what is the "sample size" row in the table?

Reply:
We hope the previous changes in the methods section have clarified the meaning of "100 simulations". Please see changes listed above. Simulations consist of sets of clusters. The sample size refers to the number of samples taken from a single cluster. We have changed the metric names used in the two results tables which report this metric for clarity, see below

Relevant changes in the manuscript:

Metric	Median	[2.5%Q, 97.5% Q]
Cluster Size	116	[53, 393]
Samples per Cluster	9	[3,34]
Latent Period (Months)	9	[4,17]
Infectious Period (Months)	3	[1,15]
$P(IS^{*})$	0.69	[0.64, 0.73]
$P(HIV^{+})$	0.53	[0.49,0.56]
$P(IS\|HIV^{-})$	0.77	[0.71, 0.81]
$P(IS\|HIV^{+})$	0.61	[0.57, 0.67]
$P(HIV^{+}\|IS = N)$	0.65	[0.58, 0.71]
$P(HIV^{+}\|IS = Y)$	0.48	[0.43, 0.52]
Tree Height (Years)	5.92	[2.59, 7.42]

Table 3: **Simulation Summary Statistics for 8 Year Simulation.** Summary statistics of simulated infectious disease outbreaks from 100 simulations where probability of transmission given contact for hosts without HIV was 1.75 times larger than for hosts with HIV. *Infection Source - transmitting an infection to at least one other host

	5 SNP Analysis	10 SNP Analysis
Participants	145	377
Clusters	21	46
Mean Samples per Cluster	7	8.43
Mean Tree Height (Years)	6.86	8.27
Infection Sources	16	43
OR and 95% CI (TP + GLM)	0.96 [0.33, 2.72]	1.03 [0.55, 1.95]
OR and 95% CI (TP + ME1)	0.88 [0.04, 17.4]	1.10 [0.18, 6.76]
Mean OR and 95% CI (TP + ME2)	0.80 [0.20, 2.90]	0.96 [0.36, 2.51]

Table 4: Summary of analyses using five and ten SNP cutoffs. Mean tree height refers to the height of timed phylogenetic trees in years. TP + GLM refers to a statistical pipeline where infection source labels are first generated using `Transphylo` and then used as response variables in a generalized linear model to calculate an odds ratio. The odds ratio is the odds ratio for the probability of being an infection source given the infection recipient is living without HIV as compared to infection recipients living with HIV.

Line 247: It seems like the "proportion reject" measure is simply the power. If so, it would be much simpler to refer to it as such throughout the text.

Reply:
Statistical power refers to the ability of a hypothesis test to reject the null hypothesis, given that the alternative hypothesis is true. In our paper we track the proportion of times that a hypothesis test rejects the null hypothesis that the true odds ratio is one. However, in one of our simulations, the true odds ratio is in fact one, i.e., the alternative hypothesis is false. Thus, we are not measuring statistical power in this scenario, and felt it best to use a more general name which describes what we are measuring across scenarios.

Figure 2: It is strange to have 4/7 when all of the other values are decimals. This also true in the supplemental figures and tables and in various points in the text. Though a 1.75 decrease expressed in decimal form would not be exact, but it would be preferrable to combining decimals and fractions in this way.

Reply:
We have changed the notation so that the 4/7 setting is now called the 0.57 setting. The changes were made to Figure 2, Table A1 and Figure A1, as well as to all places in the text which refer to the 4/7 setting. The revised Figure 2 is included below.

Relevant changes in the manuscript:

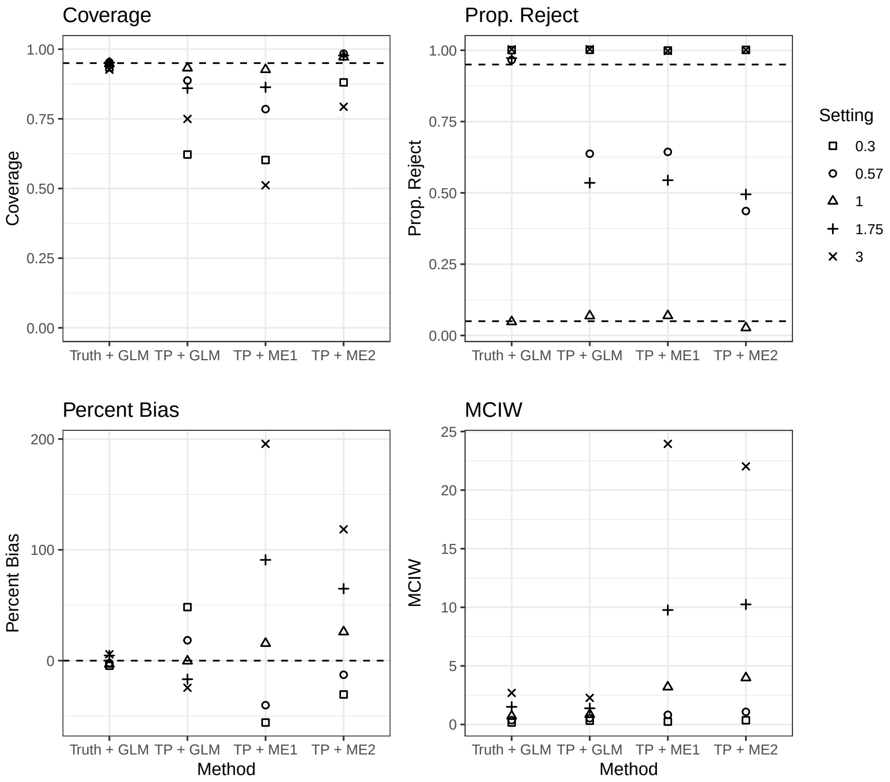

Figure 1: Operating characteristics of statistical pipelines in primary simulation settings. Truth + GLM is a reference pipeline where the true infection source labels are used as response variables in a generalized linear model. TP + GLM is a pipeline where infection source labels generated by `TransPhylo` are used as response variables in a generalized linear model. TP + ME1 is a pipeline where infection source labels are generated by `TransPhylo` and used as an input into a model from the `SAMBA` package, which allows for false positives. TP + ME2 again uses labels generated by `TransPhylo` as response variables into a model which allows for both false positives and false negatives. The settings denote different simulation settings, each value describes the true ratio of the probability of transmission given contact for cases living without HIV to cases with HIV. I.E., 0.57 means that the probability of transmission given contact for hosts without HIV was 1.75 times as small as the probability of transmission given contact for hosts with HIV. Coverage refers to proportion of simulations where 95% confidence intervals captured the true odds ratio. Prop. Reject refers to the proportion of simulations where a null hypothesis that the true odds ratio was one would be rejected, assuming significance level of 5%. Percent bias is bias divided by the true odds ratio, MCIW is mean confidence interval width.

Paragraph starting with line 268: This paragraph is hard to follow as it presents so many results of different metrics, scenarios, and methods. It would be much easier to understand the results if there was a table including all of these results and then the paragraph highlighted the important ones presenting far fewer values.

Reply:
We agree this paragraph is confusing. We feel that the results are best reported using Figure 3 (shown below), and with a less number-heavy paragraph highlighting the most relevant results from the figure.

Relevant changes in the manuscript:

The results of our secondary set of simulations (along with the original results from the 1.75 setting) are displayed in Figure 5. For the TP + GLM pipeline, increasing the percent of hosts sampled from 16% to 32% (Double Sampling Density) led to increased coverage and increased proportion of scenarios where a null hypothesis that the odds ratio was one was rejected. Quadrupling the percent of hosts sampled to 64% (Quad Sampling Density) led to a decrease in coverage compared to both the default and the Double Sampling Density simulation, although the proportion reject remained comparable with Double Sampling Density simulation, and the bias decreased. Increasing the sampling window to all but the first year of the outbreak slightly decreased coverage and increased proportion reject. Doubling the number of clusters analyzed from fifty to one hundred (Increase Sample Size) led to the lowest coverage of all settings but the highest proportion reject. For the two measurement error pipelines, the proportion reject improved in a similar manner as in the TP + GLM pipeline. However, the coverage changed in a different manner, with both the Increase Sample Sizes and the Increase Sampling Window having substantially lower coverage than the default setting.

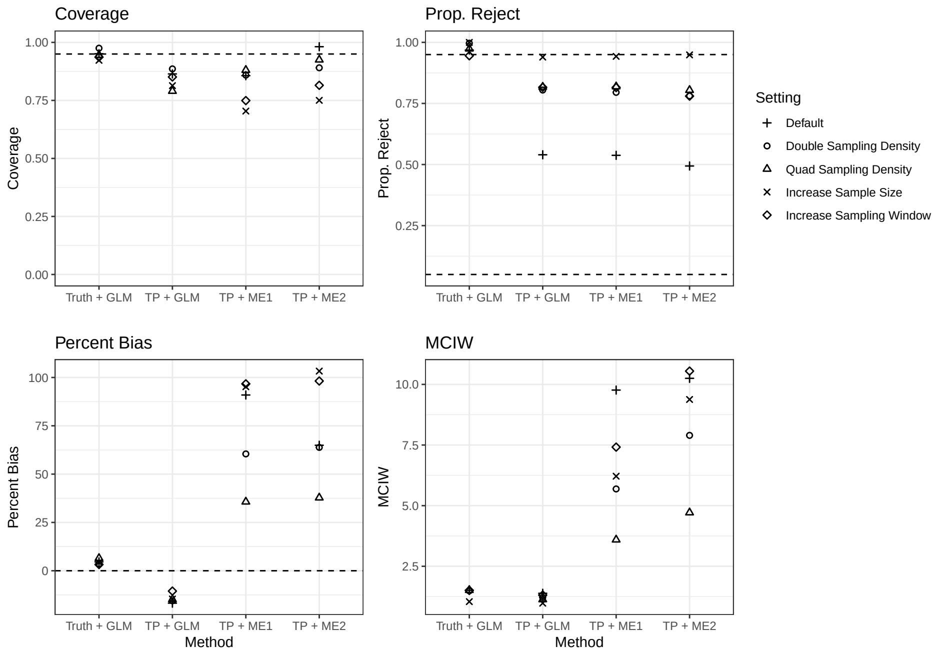

Figure 2: Operating characteristics of statistical pipelines in secondary simulation settings. See Figure 4 for x-axis and y-axis labels. The settings denote different simulation settings. For all simulations the probability of transmission given contact for cases living without HIV was 1.75 times as large as the probability of transmission given contact for cases living with HIV. Default refers to the simulation settings used in the primary simulation setting. In the Double Sampling Density setting percent of active cases sampled was doubled from 16% to 32%. In the Quad Sampling Density setting percent active cases sampled was quadrupled to 64%. In the Increase Sample Size setting the number of clusters sampled from was doubled from 50 to 100. In the Increase Sampling Window the sampling window was changed from the last three years of the simulation to the last seven years.

Additionally, in the methods one of the scenarios is described as testing an increase in the sample size (which is done by increasing the number of clusters). Here though it is described as increasing the number of clusters. It would be clearer to simply discuss it in terms of increasing sample size as the methods clarifies exactly how that is done.

Reply:
We have changed the simulation setting name to "Increase Sample Size". This changed label names in Figure 3, Appendix Figure 1 and Appendix Table 1, as well as in multiple places in the text. We will again refrain from placing them all below, given the number of minor changes involved.

Lines 299-302: This sentence is unclear.

Reply:
We have rewritten the sentence for clarity, see below.

Relevant changes in the manuscript:

Results

For our analysis, we excluded clusters with less than four sequences. We also excluded clusters with little genetic variation by excluding clusters where there were less than two times the number of sequences in the cluster minus two varying sites

---

Line 340: It would be interesting to know this value in the simulations

Reply:
The value in question is the percent of sampled cases which were identified as infection sources. We agree this would be interesting to report for a simulation as well. We have added this metric to Table 1, which describes characteristics of the 1.75 simulation.

Relevant changes in the manuscript:

Results

Metric	Median	[2.5%Q, 97.5% Q]
Cluster Size	116	[53, 393]
Samples per Cluster	9	[3,34]
Latent Period (Months)	9	[4,17]
Infectious Period (Months)	3	[1,15]
$P(IS^{*})$	0.69	[0.64, 0.73]
$P(HIV^{+})$	0.53	[0.49,0.56]
$P(IS\|HIV^{-})$	0.77	[0.71, 0.81]
$P(IS\|HIV^{+})$	0.61	[0.57, 0.67]
$P(HIV^{+}\|IS = N)$	0.65	[0.58, 0.71]
$P(HIV^{+}\|IS = Y)$	0.48	[0.43, 0.52]
Tree Height (Years)	5.92	[2.59, 7.42]
% TP Labeled IS	19.47	[17.11, 22.35]

Table 5: **Simulation Summary Statistics for 8 Year Simulation.** Summary statistics of simulated infectious disease outbreaks from 100 simulations where probability of transmission given contact for hosts without HIV was 1.75 times larger than for hosts with HIV. *Infection Source - transmitting an infection to at least one other host

---

Figure 4: Dangling sentence at the end of the caption

Reply:
We have revised the sentence, see below.

Relevant changes in the manuscript:

Results

The plot was made using (4)

Line 377: This sentence is unclear.

Reply:
We agree and have removed it. The original removed sentence is reported below.

Relevant changes in the manuscript:

Discussion

REMOVED: In contrast, we found that this pipeline had low probability of false positives for detecting an association.

Paragraph starting with line 394: In light of this discussion of sampling it would add substantially to the usefulness of this paper if there was an additional simulation scenario where the sampling proportion was very high. This would show if these methods perform better in this setting and therefore if other investigators believe that they have a very high sampling proportion they can use them. Or if the methods still do not perform well in this setting, that heightens the need for caution.

> ***Reply***:
> *Thank you for this suggestion. We have added an additional simulation to our secondary simulations where the sampling proportion is now 0.64, quadrupling the sampling proportion used in our primary simulation settings. As expected, the increased sampling proportion led to increased sensitivity for* `TransPhylo`*. Less intuitively, this increased sensitivity led to decreased coverage for the TP+GLM pipeline as compared to the simulation where we only doubled the sampling proportion. The decrease in coverage appears to be caused by overall narrower confidence interval widths. We speculate these narrower confidence intervals are a consequence of the proportion of cases who are labeled as infection sources. Essentially, as the proportion of infection sources nears fifty percent, we expect the estimated standard errors to narrow, because it is easier to estimate probabilities closer to 0.5. Our particular result is almost certainly dependent on the true effect size, that is, we would not expect that for all uses of this pipeline, a sampling proportion of 0.64 would decrease coverage as compared to a sampling proportion of 0.32. These results have caused us to rewrite the discussion in favor of much more caution when using these pipelines, as the effects of increasing the sampling proportion are unpredictable.*

Relevant changes in the manuscript:

Methods

To further investigate the consequences of increasing sampling density, we then increased the sampling proportion from 16% to 64%, again keeping the sample size approximately similar using only 13 clusters.

Quadrupling the percent of hosts sampled to 64% (Quad Sampling Density) led to a decrease in coverage compared to both the default and the Double Sampling Density simulation, although the proportion reject remained comparable with Double Sampling Density simulation, and the bias decreased

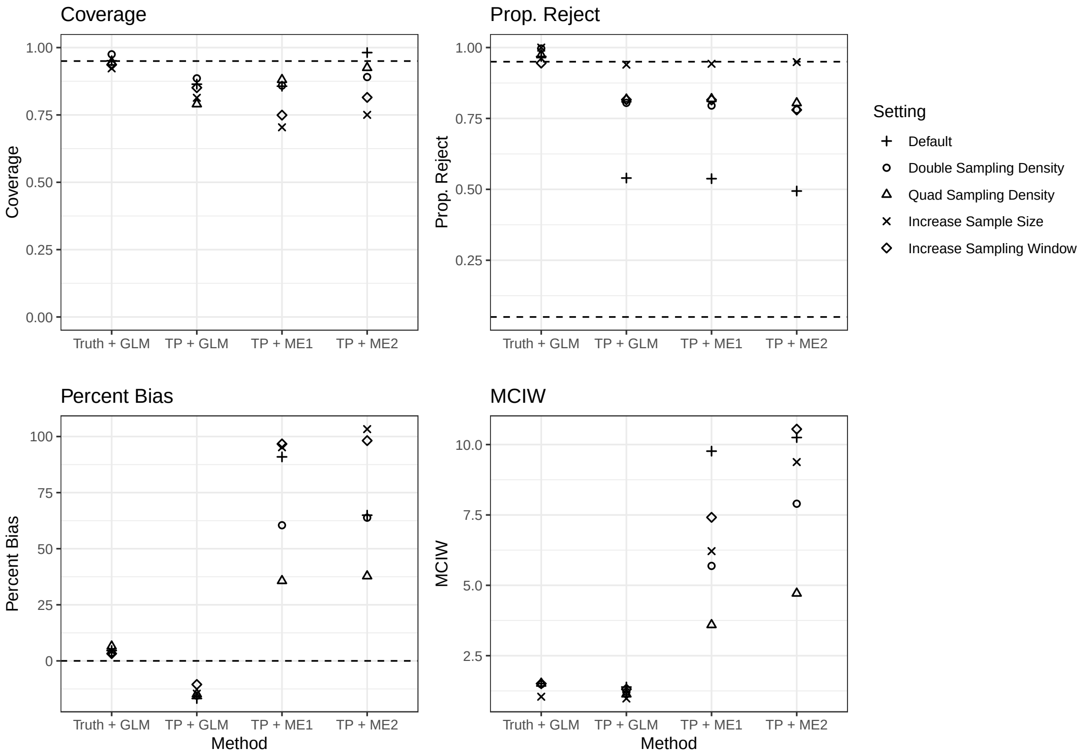

Figure 3: Operating characteristics of statistical pipelines in secondary simulation settings. See Figure 4 for x-axis and y-axis labels. The settings denote different simulation settings. For all simulations the probability of transmission given contact for cases living without HIV was 1.75 times as large as the probability of transmission given contact for cases living with HIV. Default refers to the simulation settings used in the primary simulation setting. In the Double Sampling Density setting percent of active cases sampled was doubled from 16% to 32%. In the Quad Sampling Density setting percent active cases sampled was quadrupled to 64%. In the Increase Sample Size setting the number of clusters sampled from was doubled from 50 to 100. In the Increase Sampling Window the sampling window was changed from the last three years of the simulation to the last seven years.

Discussion

We recommend carefully considering both the sampling scheme of any future studies using surveillance data when conducting research using this pipeline, as well as the plausible effect sizes researchers wish to detect.

We found that changes to the sampling scheme could improve frequentist properties of the TP + GLM pipeline. For example, doubling the number of clusters under the 1.75 setting increased the proportion of null hypotheses rejected to 0.94 (Figure 5). However, just increasing the overall sample size did not improve coverage of confidence intervals. While increased sample size decreased variance, and thus confidence interval width, it did not affect bias. In contrast, doubling the sampling density and increasing the sampling window both decreased bias and mean confidence interval width by a small amount. This led to improvements in proportion of null hypotheses rejected, and marginal improvements in coverage (increased sampling density) or only marginal losses in coverage (increased sampling window). The reduction in bias likely follows from improvements in the sensitivity of `TransPhylo` under these settings (Figures 8–9). Less intuitively, we found that quadrupling the sampling density actually decreased the coverage due to narrower confidence intervals, even though the sensitivity of `Transphylo` improved. The bias decreased, as we would expect, but the mean confidence interval width decreased so much that coverage dropped. We speculate this is a consequence of the proportion of individuals labeled as infection sources. As this proportion nears 50%, we expect the estimated standard errors to narrow, because it is easier to estimate probabilities closer to 0.5. Our particular result is almost certainly dependent on the true effect size, that is, we would not expect that for all uses of this pipeline, a sampling proportion of 0.64 would decrease coverage as compared to a sampling proportion of 0.32. Our results suggest the behavior of this pipeline is unpredictable, a higher sampling density will not automatically result in improved coverage. When exact estimation of the effect size is important, we urge great caution when using this pipeline. The improved performance under the increased sampling window suggests there may be room for improvement in the statistical model being used. Currently, `TransPhylo` assumes that the sampling proportion is constant throughout the period of the outbreak, while in our simulation scenarios sampling only took place in a fixed number of years of an outbreak (as it often would in a real life surveillance study). Future work could focus on changing this assumption in a `TransPhylo`-like model which would better match the the model likelihood to the data generation process. Though, again, we would urge caution, as improving the sensitivity of `TransPhylo` can lead to non-intuitive pipeline performance.

Line 418: Watch the capitalization of TransPhylo throughout this paragraph.

Reply:
Thank you. We have corrected the capitalization throughout the manuscript.

A.1.1: This paragraph presents many parameters with no references or justification as to how they were chosen. As the parameters are fundamental to the simulation analysis, some more detail as to the rational of the choices is warranted.

> ***Reply***:
> *We agree, and have updated the paragraph with additional details and rationales for parameter choices.*

Relevant changes in the manuscript:

Incubation periods for tuberculosis are estimated to be between a few months to two years in duration, with limited instances of longer duration (5). Based on this, the latent period for each simulated individual, during which the individual could neither leave nor transmit infection to others, was drawn from a gamma distribution with shape parameter 6.75, rate parameter 0.75 so that the majority of individuals had latent period of less than 2 years, with a mean duration of 9 months. The latent period was considered to be over at the first time step greater than or equal to the latent period length. For simplicity, individuals who are infected, but never progress to infectious TB are ignored. The probability of exiting the simulation was drawn from a beta distribution with $\alpha = 1/0.11$ and $\beta = 3/.11$ so that the mean probability was 0.25 (after the latent period was over). A mean probability of 0.25 translates into an average of three months of time during which an individual is actively infectious. For this distribution, we assumed that individuals become infectious when they start coughing and become non-infectious shortly after diagnosis and treatment. We previously found that 50% of TB diagnosis and treatment occurred within 1 month and 80% within 2 months of cough onset among people with TB in Botswana (6). We assumed a long tail for delayed diagnosis and treatment for TB (7). We used a discrete population structure with two locations (A and B) to create our two classes of cases. Individuals had the chance to move exactly once from their starting location, and the probability of moving from A to B was 0.53, while the probability of moving from B to A was 0.47. This resulted in (in general) 53% of cases staying in location B, while 47% of cases stayed in location A. These numbers were chosen to reflect the empirical proportion of those living with HIV found in our data. Number of contacts per time step was drawn from a normal distribution with mean 20 and standard deviation 5. A contact survey found people in Zambia and South Africa found participants had about four close contacts per day (8). A meta-analysis of contact surveys found average daily contacts globally was about 9 contacts per day (9). We would expect that in a month, individuals see the same people multiple times, so that daily contacts don't translate to monthly contacts. This is hard to quantify rigorously, but we felt an average of 20 unique contacts a month was not unrealistic. In practice, the number of contacts mostly controls the size of the outbreak, because new infections are not dependent on current infections in our simulations. The normal random variable was then rounded and the absolute value used as the number of contacts. The basic reproduction number was chosen to be 1.18, that is, on average, individuals infect 1.18 other individuals. This number has been shown to vary substantially in different settings, ranging from 0.24 to 4.3 in previous studies.(10) We chose the reproduction number of 1.18 to mimic a disease which is spreading steadily but where the slope of incidence is not growing incredibly quickly, which reflects TB spread in high prevalence countries such as Botswana. We can write the basic reproduction number as a function of the average total number of contacts during the infectious period multiplied by the probability of transmission given contact.

Appendix

In mathematical terms:

$$\texttt{Basic Reproduction Number} = \textbf{Average Total Contacts}* \\ (0.47 * P(\texttt{Transmission|Contact in A})+ \\ (0.53 * P(\texttt{Transmission| Contact in B})).$$

Thus, probability of transmission given contact for each location was calculated beginning from a starting point of having the base reproduction number be 1.18, the mean number of contacts be 20, and the mean number of actively infectious time steps be 3. The exact values changed between simulation settings, depending on the desired ratio for probability of transmission given contact for the two classes of individuals.

A.1.1: The sentence "Probability of transmission given…" is unclear and should be explained in more detail.

Reply:
We have expanded on this in more detail, see below.

Relevant changes in the manuscript:

Appendix

The basic reproduction number was chosen to be 1.18, that is, on average, individuals infect 1.18 other individuals. We can write the basic reproduction number as a function of the average total number of contacts during the infectious period multiplied by the probability of transmission given contact. In mathematical terms:

$$\texttt{Basic Reproduction Number} = \textbf{Average Total Contacts}* \\ (0.47 * P(\texttt{Transmission|Contact in A})+ \\ (0.53 * P(\texttt{Transmission| Contact in B})).$$

Thus, probability of transmission given contact for each location was calculated beginning from a starting point of having the base reproduction number be 1.18, the mean number of contacts be 20, and the mean number of actively infectious time steps be 3. The exact values changed between simulation settings, depending on the desired ratio for probability of transmission given contact for the two classes of individuals.

A.1.1: The sentence "Note that due..." is concerning. First of all, it is not clear exactly what ratio is being referred to though it can be assumed that it is the increase in risk of being an infection source when HIV+ in the scenario when it should be 3.00. Although it is true that using a value of 3.02 would almost certainly have almost no impact on the results, it is unprofessional to publish a paper with an error. If the authors intend to use a ratio of 3.00, they should correct the results.

Reply:
To provide a little more context, in the case of the 3.02 simulation setting, we ran one million simulations in order to estimate the true odds ratio with acceptably low Monte Carlo standard error. This process took hundreds of hours of computing time. Given this context and because this will have no meaningful impact, we have kept the ratio of 3.02. We have changed the methods section to make it explicit that we are testing a ratio of 3.02 not 3.

Relevant changes in the manuscript:

Methods

The settings were: probability of transmission given contact was 3.02 times higher among people living without HIV compared to that among people with HIV, 1.75 times higher, equal, 1.75 times lower, or 3.33 times lower. All other parameters were identical for the two classes of cases. For the 3.02 setting, we will round down and refer to this as the 3 setting for the duration of the paper.

Appendix

REMOVED: Note that due to coding errors, we actually tested a ratio of 3.02, rather than a ratio of 3. We felt this was not worth correcting given the negligible difference in ratios.

A.2: Clarify what "3" setting is

Reply:
We have rewritten the sentence for clarity.

Relevant changes in the manuscript:

We attempted using BCa Bootstrap as an additional step in the TP + GLM pipeline for the primary simulation setting where the probability of transmission given contact was 3.02 times higher for people living without HIV compared to people with HIV.

Table A1: It is not clear why the authors truncated and not rounded the values used in the different settings. Although it makes no meaningful difference as they stated, there will always be a decision between two values and therefore rounding seems to be the best option unless there is a justification for truncation.

***Reply**:*
We have switched to rounding the point estimate reported in Table A1 (Now Table A3) to the nearest hundredths place. Note that this meant we changed the true Odds Ratios for the 0.3, 1.75 and 3 simulation settings, as well as a secondary simulation settings which used the 1.75 odds ratio. The value used for the 0.57 and 1 simulation settings would be the same under the new rounding. This changed the results somewhat for the primary and secondary simulations, but overall results remained the same as before.

Relevant changes in the manuscript:

Then, for each version of our pipeline and each simulation setting, we calculated frequentist coverage (proportion of simulations for which a 95% confidence/credible interval contained the true parameter–ideally it should be 0.95), proportion reject: the proportion of times that a null hypothesis that the odds ratio was one was rejected (with significance level 5%), percent bias (bias divided by the true odds ratio), and mean confidence interval width of 95% confidence intervals. Figure 4 shows the results from the primary set of simulations. The four pipelines tested for each simulation were: a reference pipeline where true infection source labels were known and used as inputs into a generalized linear model (Truth + GLM), a pipeline where `TransPhylo` labels were used as input into a generalized linear model (TP + GLM), a pipeline where `TransPhylo` labels were used as input into SAMBA (TP + ME1) for misclassification adjustment and a pipeline where `TransPhylo` labels were used as inputs in our Bayesian model for misclassification adjustment (TP + ME2). Across simulation settings, the Truth + GLM pipeline had coverage around 0.95, proportion reject above 0.95 (except in the case when the odds ratio was 1), percent bias near 0 and confidence interval width ranging from 0.17 to 2.7. For the TP + GLM pipeline, coverage varied depending on the simulation setting, ranging from 0.62 to 0.93. When the true odds ratio was not one, TP + GLM had proportion reject ranging from 0.54 to 1. Percent bias ranged from -16.76% to 48.34%, mean credible interval width was 0.33 to 2.27. The TP + ME1 pipeline had coverage and proportion reject similar to TP + GLM, with percent bias ranging from -55.83% to 195.66% and MCIW between 0.25 and 23.94. Finally, the TP + ME2 pipeline had coverage ranging from 0.78 to 0.98, proportion reject ranging from 0.44 to 1, percent bias from -30.52% to 119.89% and MCIW from 0.35 to 22.24.

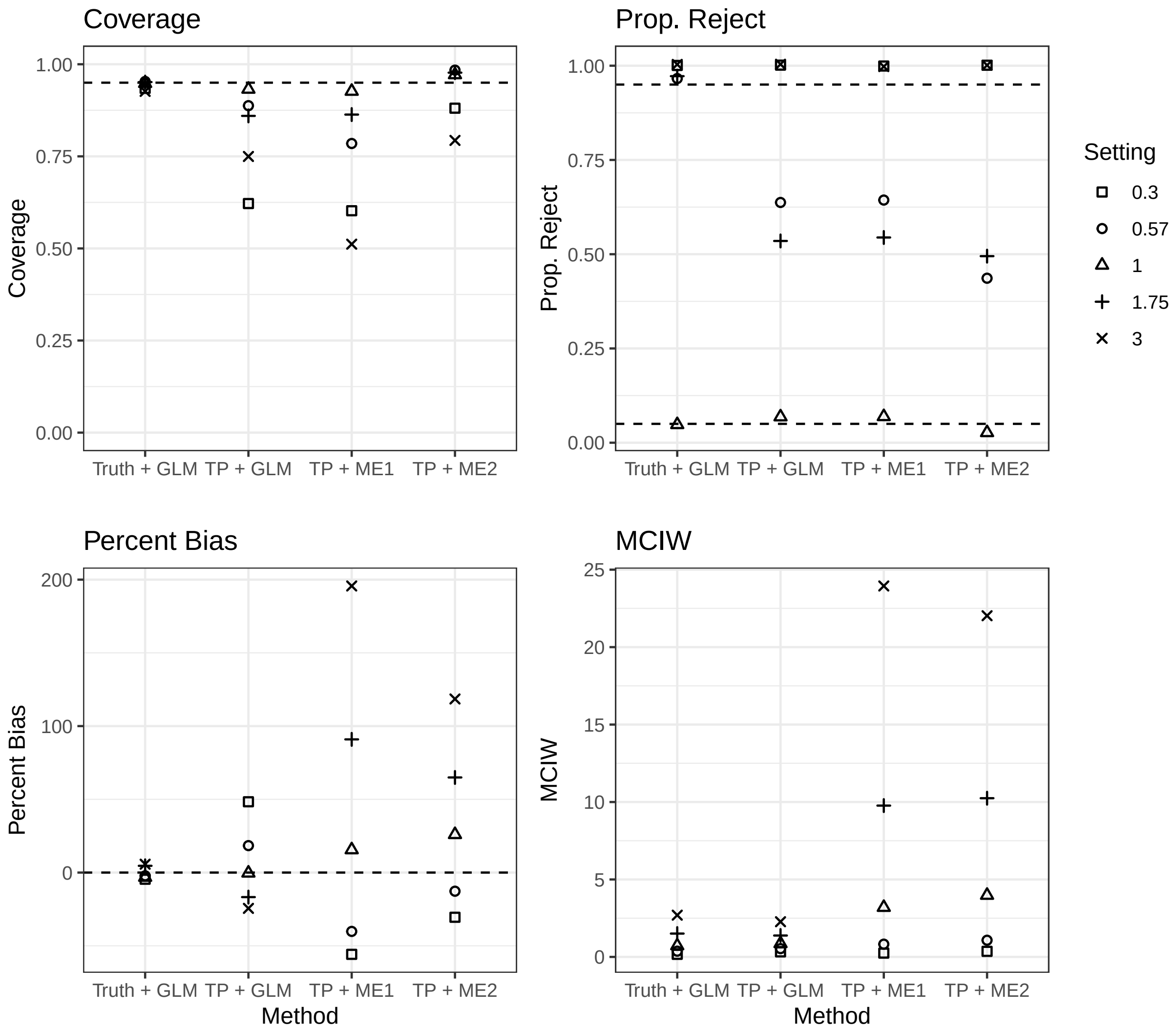

Figure 4: Operating characteristics of statistical pipelines in primary simulation settings. Truth + GLM is a reference pipeline where the true infection source labels are used as response variables in a generalized linear model. TP + GLM is a pipeline where infection source labels generated by `TransPhylo` are used as response variables in a generalized linear model. TP + ME1 is a pipeline where infection source labels are generated by `TransPhylo` and used as an input into a model from the `SAMBA` package, which allows for false positives. TP + ME2 again uses labels generated by `TransPhylo` as response variables into a model which allows for both false positives and false negatives. The settings denote different simulation settings, each value describes the true ratio of the probability of transmission given contact for cases living without HIV to cases with HIV. I.E., 0.57 means that the probability of transmission given contact for hosts without HIV was 1.75 times as small as the probability of transmission given contact for hosts with HIV. Coverage refers to proportion of simulations where 95% confidence intervals captured the true odds ratio. Prop. Reject refers to the proportion of simulations where a null hypothesis that the true odds ratio was one would be rejected, assuming significance level of 5%. Percent bias is bias divided by the true odds ratio, MCIW is mean confidence interval width.

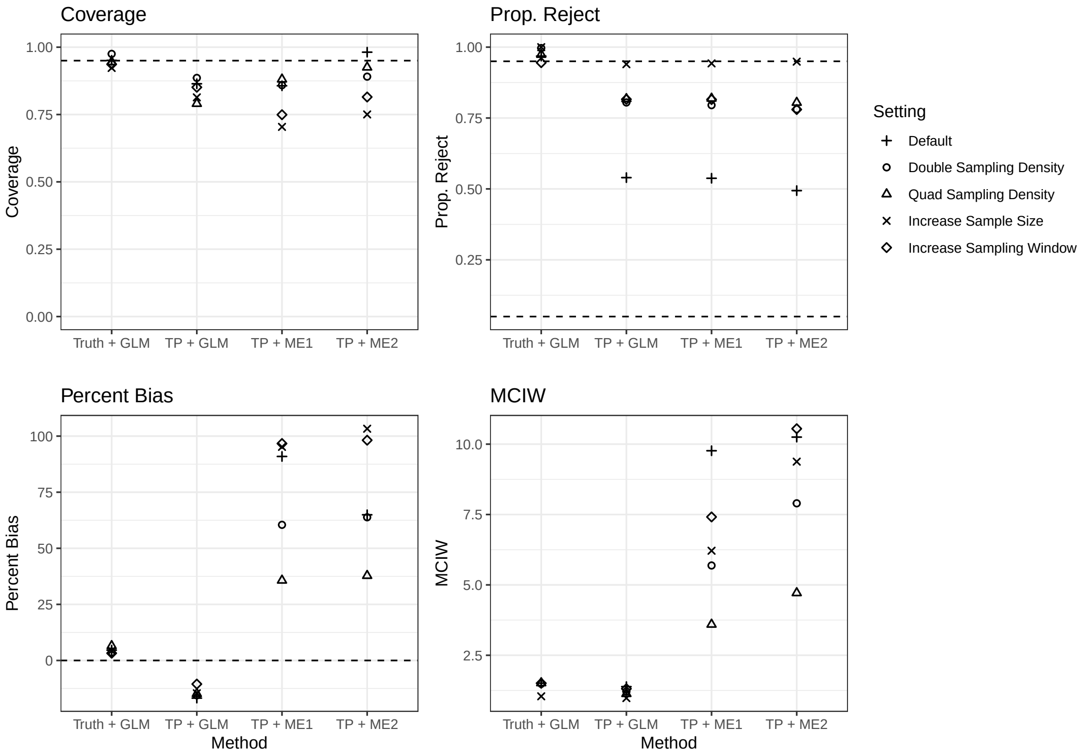

Figure 5: Operating characteristics of statistical pipelines in secondary simulation settings. See Figure 4 for x-axis and y-axis labels. The settings denote different simulation settings. For all simulations the probability of transmission given contact for cases living without HIV was 1.75 times as large as the probability of transmission given contact for cases living with HIV. Default refers to the simulation settings used in the primary simulation setting. In the Double Sampling Density setting percent of active cases sampled was doubled from 16% to 32%. In the Quad Sampling Density setting percent active cases sampled was quadrupled to 64%. In the Increase Sample Size setting the number of clusters sampled from was doubled from 50 to 100. In the Increase Sampling Window the sampling window was changed from the last three years of the simulation to the last seven years.

Setting	Point Estimate	Standard Error	99% CI	Value Used
0.3	0.2487	0.00066	[0.2473, 0.2499]	0.25
0.57	0.5205	0.0013	[0.5178, 0.5232]	0.52
1	1	NA	NA	1
1.75	1.9381	0.00176	[1.9345, 1.9415]	1.94
3	3.5298	0.0014	[3.5267, 3.5327]	3.53

Table 6: Monte Carlo estimates for the true Odds Ratios of probability of being an infection source used in simulations. Value Used denotes the value used in the simulations when calculating frequentist metrics.

Figure A1: This should be two separate figures as the overlapping color schemes make it difficult to interpret. Additionally, the color scheme for the five settings should be more carefully chosen as it is difficult to differentiate between orange and yellow (though the dots are currently overlapping they might not be if the figures were split and enlarged).

Reply:
We have split the two figures as suggested, and reordered the colors to improve contrast within the two plots.

Relevant changes in the manuscript:

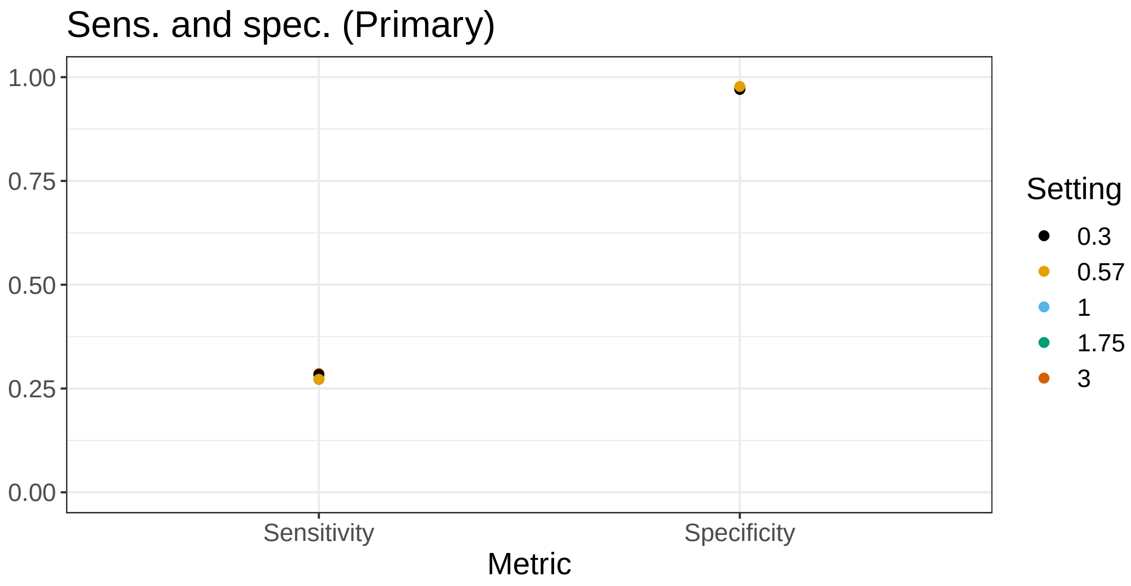

Figure 6: Point estimates of sensitivity and specificity of identifying infection sources based on `TransPhylo` estimates. Settings refer to simulation settings from the primary simulations.

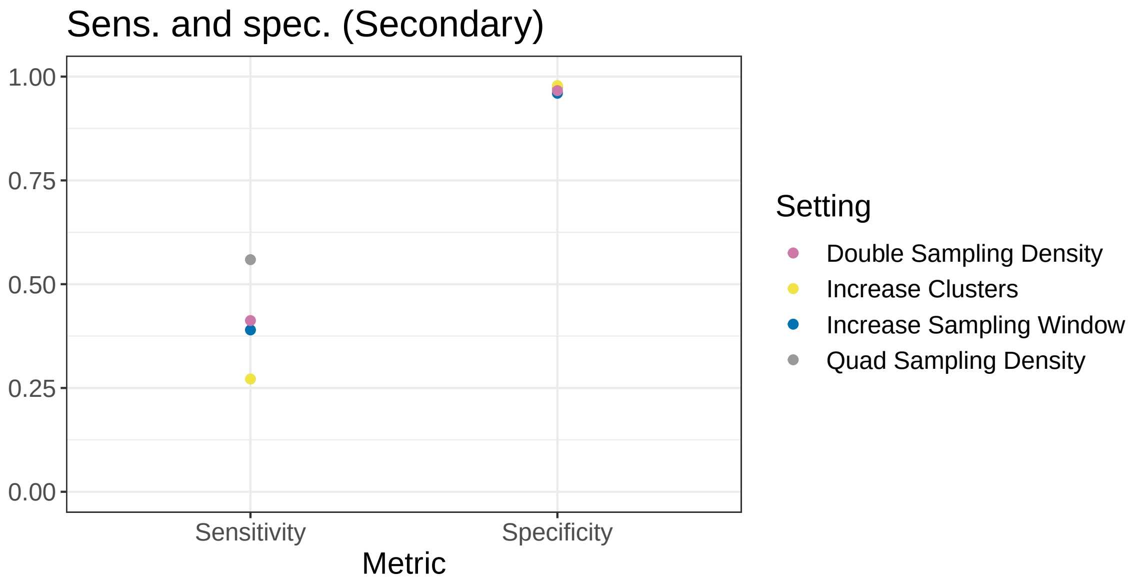

Figure 7: Point estimates of sensitivity and specificity of identifying infection sources based on `TransPhylo` estimates. Settings refer to simulation settings from the secondary simulations.

Comments from Reviewer 3 The main revision I would like to see in this manuscript is to describe the genomic diversity of the clusters used in the study and review the performance of the tested approach in the relation to this diversity, along with the sampling proportion. It would be interesting to see if there are any cluster-level differences in the ability to correctly associate HIV infection and transmission source when considering the genomic diversity of a cluster. In general, the lower the diversity in a cluster, the greater the phylogenetic uncertainty where relationships between identical or near identical sequences must be inferred from the sequence data and collection date alone. This is particularly relevant in this analysis that has used a single MCC tree for each cluster to run the transmission inference, and where a relatively narrow time interval (3 years) is used. I think it would be useful to see some measure of the genomic diversity in clusters and some discussion on whether this may influence the main results of the accuracy of the transmission inference in simulated and real-world data.

Reply:
We agree genomic diversity plays a role in how well phylogenetic trees can be reconstructed from genomic data, and that this would have downstream impacts on on the performance of the rest of the statistical pipeline. However, how the pipeline would be affected cannot be addressed using the tools utilized in this study. The reason for this is that we used the simulation engine `nosoi`(11), which generates timed phylogenetic trees, and does not generate individual genetic sequences. In practice, what this means is that our study assumes the most optimistic case; that there is sufficient genetic diversity to completely correctly reconstruct a timed phylogenetic tree. We would expect the performances of the pipelines we tested to decline in settings where the timed phylogenetic trees were not completely correct. It would be useful in future studies to also evaluate these pipelines using simulation engines which generate sequence data. We have expanded the discussion to further highlight these points.

Relevant changes in the manuscript:

Discussion

In practice, these phylogenies must also be estimated from sequence data. Depending on the genetic diversity of available sequences, there may also be errors when creating the timed phylogeny. The errors in reconstruction of phylogenies could propagate further down the statistical pipeline, reducing its overall performance.

Limitations

Finally, our study assessed pipeline performance in the best case scenario: when timed phylogenetic trees are perfectly accurate. However, in the real world, phylogenetic trees must first be constructed from sequence data, a process which may affect pipeline performance. Conducting assessments of these pipelines using simulation engines which simulate sequence data is a useful area of future research.

It would be worthwhile to make the distinction here between low-incidence and high-incidence setting in the application of this work, although this has been addressed in the discussion. The authors estimate that the sampling density in their real-world data from Botswana is 16%. This value can be higher in low-incidence or well-resourced settings.

Reply:
We agree this could be emphasized more, and have expanded a paragraph in the introduction to highlight this point.

Relevant changes in the manuscript:

Introduction

While passive disease surveillance could capture a significant portion of infectious disease incidence, we expect a meaningfully smaller proportion of cases will be sampled as compared to an outbreak investigation study. It is worth pointing out that we expect the proportion of cases sampled to heavily depend on the resources of a public health system, as well as the prevalence of the disease being studied. In well resourced public health systems, the difference between passive surveillance and outbreak investigation may be small. Still, the performance of many of the transmission reconstruction methods in the context of passive surveillance is not at all clear.

Could the authors report any studies or empirical data, or explain the rationale, to support their parameter choices for the simulated data, notably the latent (mean 9 months) and infectious period (mean = 3 months).

Reply:
We have updated the appendix with additional discussion and citations to give more insight into these modeling choices.

Relevant changes in the manuscript:

Appendix

See response to reviewer two.

Again, choice of prior parameter choices for TransPhylo, particularly the generation time distribution with mean of 1 year, should be justified. Also were these TransPhylo parameter estimates used for both

Reply:
We are viewing the generation time distribution as the sum of the latent period distribution and the infectious period distribution. Thus the justification for the mean generation time is the same as for the mean latent and infectious periods, see previous reply for more details. Using published estimates of the serial interval for tuberculosis of 0.57 to 1.65 years as a reference point, our mean generation interval of 1 year is reasonable.(10) TransPhylo only allows gamma distributions for the generation time, so we can't exactly mimic the distribution which is the sum of the latent and infectious period distributions, but we can at least match the mean, and choose parameters for the gamma distribution so that there is a reasonably large range of possible generation times. We chose the prior for the sampling proportion based on early simulation results under our sampling regime which is meant to resemble the sampling regime of our study setting. All other priors in TransPhylo are fixed. The same parameters were used for both simulated and real data. We have updated the appendix with additional details on these choices.

Relevant changes in the manuscript:

Note that we chose the sampling distributions to be the same as the generation time distributions. We felt this was plausible as it reflects our assumption that individuals in their latent stage would not be detected in our sampling regime. We chose to have the mean generation time be 1 year, based on our choices for the mean latent period (9 months) and the mean infectious period (3 months). Treating the mean generation time as the sum of the mean latent and mean infectious periods is a reasonable choice, see previous work on this topic by Svennson and Champredon et al. for more details (12; 13). Moreover, our mean generation time is consistent with published estimates for the serial interval for tuberculosis of 0.57 to 1.65 years.(10) See below for rationales behind our choices for the mean latent and infectious periods. The sampling prior was chosen based on early empirical results from simulations where we sampled only from the last three years in the sampling regime described in the main methods, which is meant to resemble the sampling regime in our study setting. The same parameters were used for both simulated and real data. See below for rationales behind our choices for the mean latent and infectious periods. The sampling prior was chosen based on early empirical results from simulations where we sampled only from the last three years in the sampling regime described in the main methods, which is meant to resemble the sampling regime in our study setting. The same parameters were used for both simulated and real data.

Line 223 – Were all sequences with any number of heterozygous sites excluded? This would be very strict as heterozygous sites can occur in non-mixed infections when within-host clonal diversity is present. I would suggest either excluding only sequences with an excess number of heterozygous sites or using an existing method to detect mixed infection (such as doi: 10.1186/s12864-018-4988-z). I I'm not sure it would be worth conducting the full analysis again to include these sequences as it will not likely affect the results of this study though.

Reply:
We only excluded sequences identified as having mixed populations as detected by our bioinformatics pipeline (14). Our algorithm flags sequences with at least 45–50 heterozygous sites. Those that are not classified as mixed infection remained in the analysis. We have added relevant details to this in the Methods.

Relevant changes in the manuscript:

Methods

We excluded sequences with heterogeneous reads along a base position for 45–50 SNPs or greater, which is indicative of mixed infections, leaving 1347 sequences for analysis.

Line 186: – please include the code for this new approach. Given the recommendation to use this

Reply:
All code used in this project is available on github. We have updated the methods section to include the github url, see below.

Relevant changes in the manuscript:

Methods

All code and data needed to recreate the results of this study are available at `https://github.com/igoldsteinh/kopanyo_tp_code`**.**

Line 247: – Explain the term 'frequentist coverage' and how to interpret the value. I can see this work will be of interest to epidemiologists and biologists with less statistical background so it would be helpful to include a small explanation in the text.

Reply:
We have revised the sentence for clarity, see below.

Relevant changes in the manuscript:

Results

Then, for each version of our pipeline and each simulation setting, we calculated frequentist coverage (proportion of simulations for which a 95% confidence/credible interval contained the true parameter–ideally it should be 0.95), proportion reject: the proportion of times that a null hypothesis that the odds ratio was one was rejected (with significance level 5%), percent bias (bias divided by the true odds ratio), and mean confidence interval width of 95% confidence intervals.

-Line 297 – I would suggest moving this whole section to methods rather than results.

Reply:
We have moved the suggested section (on analyzing real data) to the methods section. We will refrain from including the whole section in this letter.

Relevant changes in the manuscript: Section has been moved from results to methods.

Figure A1 – I would suggest placing the legend for each plot next to plot is relates to, rather than together as it makes it a little hard to read.

Reply:
At the suggestion of reviewer 2 we have split this figure into two separate figures. We hope that this helps improve the readability of the plots.

Relevant changes in the manuscript:

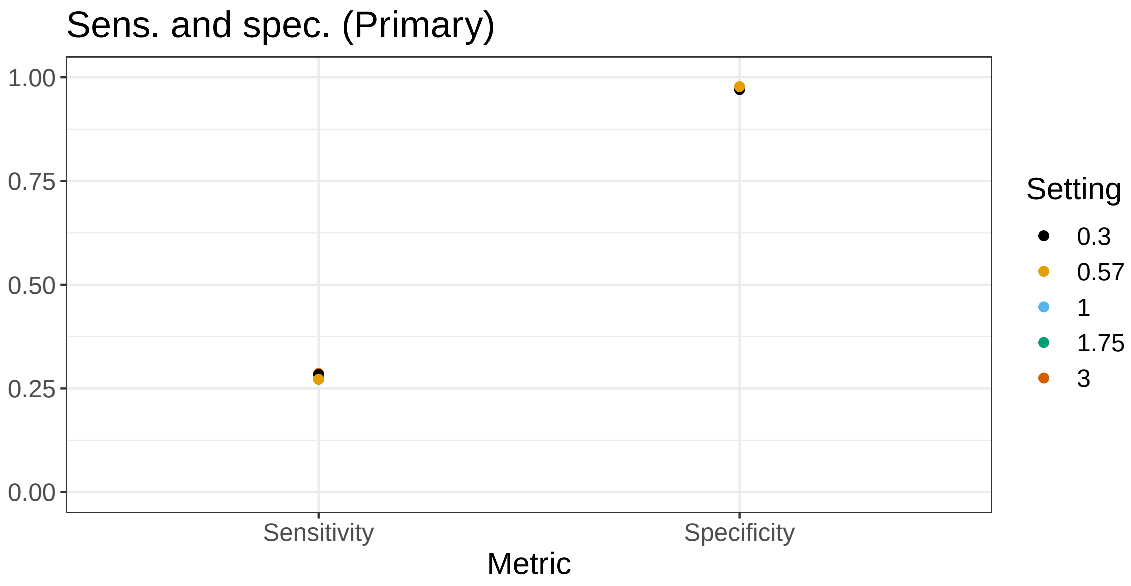

Figure 8: Point estimates of sensitivity and specificity of identifying infection sources based on `TransPhylo` estimates. Settings refer to simulation settings from the primary simulations.

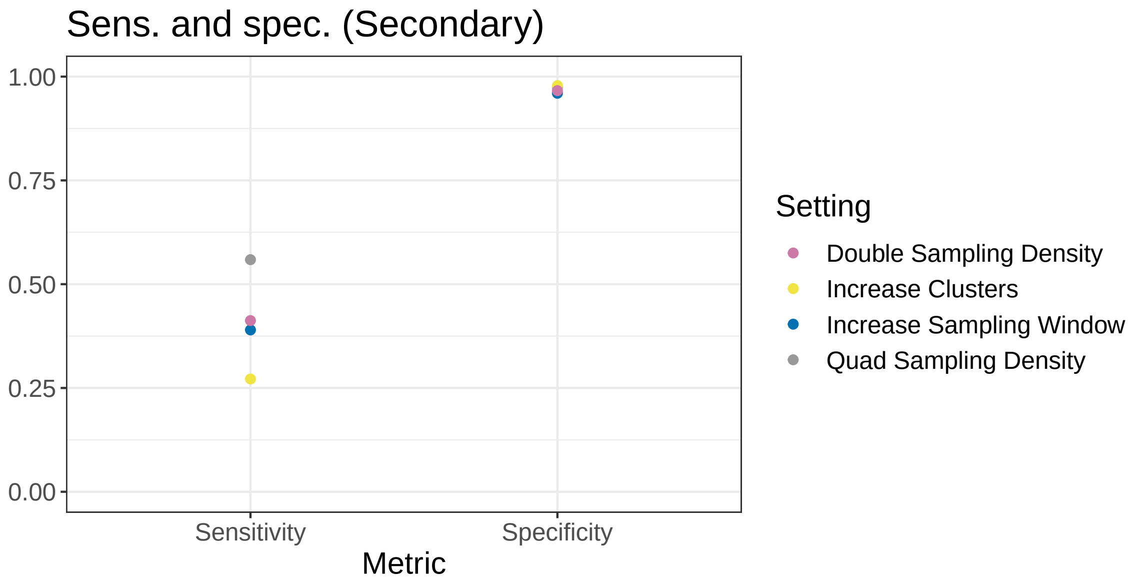

Figure 9: Point estimates of sensitivity and specificity of identifying infection sources based on `TransPhylo` estimates. Settings refer to simulation settings from the secondary simulations.

References

[1] Bouackert, Remco. Ascertainment correction/Sampling bias; 2019. Available from: https://www.beast2.org/2019/07/18/ascertainment-correction.html.

[2] Menardo F, Duchêne S, Brites D, Gagneux S. The molecular clock of Mycobacterium tuberculosis. PLoS Pathogens. 2019;15(9):e1008067.

[3] Organization WH. Global Tuberculosis Report 2020;. https://www.who.int/publications-detail-redirect/9789240013131.

[4] Yu G, Smith DK, Zhu H, Guan Y, Lam TTY. ggtree: an R package for visualization and annotation of phylogenetic trees with their covariates and other associated data. Methods in Ecology and Evolution. 2017;8(1):28–36.

[5] Behr MA, Edelstein PH, Ramakrishnan L. Revisiting the timetable of tuberculosis. Bmj. 2018;362.

[6] Molebatsi K, Wang Q, Dima M, Ho-Foster A, Modongo C, Zetola N, et al. Depression and delayed tuberculosis treatment initiation among newly diagnosed patients in Botswana;16(7):1088–1098. doi:10.1080/17441692.2020.1826049.

[7] Cox H, Dickson-Hall L, Ndjeka N, Hoog Av, Grant A, Cobelens F, et al. Delays and loss to follow-up before treatment of drug-resistant tuberculosis following implementation of Xpert MTB/RIF in South Africa: A retrospective cohort study. 2017;14(2):e1002238. doi:10.1371/journal.pmed.1002238.

[8] Dodd PJ, Looker C, Plumb ID, Bond V, Schaap A, Shanaube K, et al. Age-and sex-specific social contact patterns and incidence of Mycobacterium tuberculosis infection. American journal of epidemiology. 2016;183(2):156–166.

[9] Mousa A, Winskill P, Watson OJ, Ratmann O, Monod M, Ajelli M, et al. Social contact patterns and implications for infectious disease transmission–a systematic review and meta-analysis of contact surveys. ELife. 2021;10:e70294.

[10] Ma Y, Horsburgh CR, White LF, Jenkins HE. Quantifying TB transmission: a systematic review of reproduction number and serial interval estimates for tuberculosis. 2018;146(12):1478–1494. doi:10.1017/S0950268818001760.

[11] Lequime S, Bastide P, Dellicour S, Lemey P, Baele G. nosoi: A stochastic agent-based transmission chain simulation framework in R. Methods in Ecology and Evolution. 2020;11(8):1002–1007.

[12] Svensson A. A note on generation times in epidemic models. Mathematical Biosciences. 2007;208(1):300–311.

[13] Champredon D, Dushoff J. Intrinsic and realized generation intervals in infectious-disease transmission. Proceedings of the Royal Society B: Biological Sciences. 2015;282(1821):2015–2026. doi:10.1098/rspb.2015.2026.

[14] Kohl TA, Utpatel C, Schleusener V, De Filippo MR, Beckert P, Cirillo DM, et al. MTB-seq: a comprehensive pipeline for whole genome sequence analysis of Mycobacterium tuberculosis complex isolates. PeerJ. 2018;6:e5895.
