# Supplementary material for: Using genetic data to identify transmission risk factors: Statistical assessment and application to tuberculosis transmission
Source: PLoS Comput Biol. 2022 Dec 5;18(12):e1010696. doi: 10.1371/journal.pcbi.1010696 (PMC9754595; doi:10.1371/journal.pcbi.1010696)
Supplement: Attachment — Submitted filename: PCOMPBIOL Review.docx [file pcbi.1010696.s002.docx]

Goldstein et al. present an interesting simulation analysis exploring the estimation of risk factors of being an infection source for TB. They also include an application to TB surveillance data in Botswana. They simulated TB-like outbreaks and then inferred transmission trees from a sparse subset of those outbreaks using the TransPhylo R package. They then used the inferred infector-infectee links to estimate the effect of being HIV+ on being an infection source. They found considerable bias, low coverage, and low power to detect the true associations. Various methods to account for measurement error and changes to the parameters in the model did little to improve estimation in most settings. They therefore conclude that caution should be taken when using similar estimation pipelines to make conclusions about the effect of individual characteristics on being an infection source. Although primarily a paper with only a negative result, I think this is highly valuable to the field. Using pathogen genetic data to find risk factors of transmission is already being used and there are limited studies that explore how well it performs. However, I think that there are many places that the paper needs clarification, and the wording often makes it difficult to follow the methodology. See below for specific comments.

Introduction

Line 37: This sentence is confusing. It would probably be better to simply remove everything after “when the response…”

Lines 43-44: It isn’t clear what the “response variable” means in this context making it difficult to understand this sentence.

Lines 51-57: This is a discussion of the results and should not be in the introduction.

Methods

Lines 69-73: This sentence is hard to understand because of the use of the term “disease recipient”. The word “recipient” makes the term sounds like it is referring to the infectee in a given infector-infectee link. Therefore, a “disease recipient” can’t also be an “infection source” in a given infection event. However, it seems like the term is being used to simply mean an individual in an outbreak, i.e. a case. This is a general issue throughout the methods that makes it hard to track. I think instead of using “disease recipient” or “infection recipient” to refer to an individual in the simulation it would be better to use a generic word like simply “individual” or “case”. This would greatly aid in clarifying the methods.

Lines 104-106: It is unclear why the authors use 3 times lower and then 3.33 times higher? Why not 3 times higher? But, in the rest of the paper it seems like they did use 3 times higher, so then why does it say 3.33 times here?

Line 108 and 112: It is not clear from earlier in this section what a set of clusters would be. The description of the simulation set up seems to imply that 1 simulation = 1 outbreak = 1 cluster. What then would differentiate 100 sets of 50 clusters from 5000 simulations (in other words 5000 clusters)? This should be clarified. Similarly, the sentence “only simulations where all fifty clusters had at least two sampled individuals were accepted” is not clear in light of the previous simulation description.

Lines 116-121: Since there was a clear method for getting the value of 16% it would be helpful to provide the calculation of how to go from 54% to 16% including the values used for the number of “sequences available” and “number of estimated TB episodes” with sources.

Line 123: New sentence should start after “nosi”

Lines 136-138: Remove “in the second setting” and “in the third setting”, they only make the paragraph more confusing. It would also be very helpful to have a supplemental table that lists all of the different simulation scenarios split by primary and secondary including the effect size, number of clusters, sampling proportion, sampling window, and anything other inputs that changed between the settings.

Line 144: What are “all disease parameters”? The ones described below?

Line 147: Does “sampling prior” mean the sampling proportion prior? This is another place where a supplemental table listing all of the parameters and their prior distributions would be helpful.

Line 165: It is reverse than you would expect for HIV to be 1 when the individual is HIV- and 0 if the individual is HIV+. It doesn’t make a difference but using this coding, it would be clearer to have “HIV-“ in the equation instead of just “HIV”.

Results

Table 1: It says this is for 100 simulations. Is that 100 clusters? 100 sets of 50 clusters so 5000 clusters? This should be clarified. Also, what is the “sample size” row in the table?

Line 247: It seems like the “proportion reject” measure is simply the power. If so, it would be much simpler to refer to it as such throughout the text.

Figure 2: It is strange to have 4/7 when all of the other values are decimals. This also true in the supplemental figures and tables and in various points in the text. Though a 1.75 decrease expressed in decimal form would not be exact, but it would be preferrable to combining decimals and fractions in this way.

Paragraph starting with line 268: This paragraph is hard to follow as it presents so many results of different metrics, scenarios, and methods. It would be much easier to understand the results if there was a table including all of these results and then the paragraph highlighted the important ones presenting far fewer values. Additionally, in the methods one of the scenarios is described as testing an increase in the sample size (which is done by increasing the number of clusters). Here though it is described as increasing the number of clusters. It would be clearer to simply discuss it in terms of increasing sample size as the methods clarifies exactly how that is done.

Lines 299-302: This sentence is unclear.

Line 340: It would be interesting to know this value in the simulations

Figure 4: Dangling sentence at the end of the caption

Discussion

Line 377: This sentence is unclear.

Paragraph starting with line 394: In light of this discussion of sampling it would add substantially to the usefulness of this paper if there was an additional simulation scenario where the sampling proportion was very high. This would show if these methods perform better in this setting and therefore if other investigators believe that they have a very high sampling proportion they can use them. Or if the methods still do not perform well in this setting, that heightens the need for caution.

Line 418: Watch the capitalization of TransPhylo throughout this paragraph.

Appendix

A.1.1: This paragraph presents many parameters with no references or justification as to how they were chosen. As the parameters are fundamental to the simulation analysis, some more detail as to the rational of the choices is warranted.

A.1.1: The sentence “Probability of transmission given…” is unclear and should be explained in more detail.

A.1.1: The sentence “Note that due…” is concerning. First of all, it is not clear exactly what ratio is being referred to though it can be assumed that it is the increase in risk of being an infection source when HIV+ in the scenario when it should be 3.00. Although it is true that using a value of 3.02 would almost certainly have almost no impact on the results, it is unprofessional to publish a paper with an error. If the authors intend to use a ratio of 3.00, they should correct the results.

A.2: Clarify what “3” setting is

Table A1: It is not clear why the authors truncated and not rounded the values used in the different settings. Although it makes no meaningful difference as they stated, there will always be a decision between two values and therefore rounding seems to be the best option unless there is a justification for truncation.

Figure A1: This should be two separate figures as the overlapping color schemes make it difficult to interpret. Additionally, the color scheme for the five settings should be more carefully chosen as it is difficult to differentiate between orange and yellow (though the dots are currently overlapping they might not be if the figures were split and enlarged).
