# Supplementary material for: Using genetic data to identify transmission risk factors: Statistical assessment and application to tuberculosis transmission
Source: PLoS Comput Biol. 2022 Dec 5;18(12):e1010696. doi: 10.1371/journal.pcbi.1010696 (PMC9754595; doi:10.1371/journal.pcbi.1010696)
Supplement: Attachment — Submitted filename: Goldstein_Reviewer_Sobkowiak.docx [file pcbi.1010696.s003.docx]

**General Comments**
This study by Goldstein and colleagues reconstructs TB transmission from phylogenetic trees in simulated and real-world clusters and investigates the effect of low sampling on the ability to associate host factors with transmission source, as well as the evaluating the performance of TransPhylo when identifying source cases of transmission. This transmission reconstruction framework has been used previously to predict the effects of host factors on the risk of onward TB transmission from whole genome sequences collected as part of TB surveillance programs, where the sampling proportion of all TB cases can be relatively low compared to densely sampled outbreaks. The results of this study suggest that the inferences made in such studies on the factors that may be associated with TB transmission should be interpreted with caution if sampling density is low. This work has been well-constructed and recognises a key limitation with the interpretation of transmission networks reconstructed using phylogenetic trees. I believe this work will be of great interest to the field of TB transmission and genomic epidemiology.

**Specific Comments**
The main revision I would like to see in this manuscript is to describe the genomic diversity of the clusters used in the study and review the performance of the tested approach in the relation to this diversity, along with the sampling proportion. It would be interesting to see if there are any cluster-level differences in the ability to correctly associate HIV infection and transmission source when considering the genomic diversity of a cluster. In general, the lower the diversity in a cluster, the greater the phylogenetic uncertainty where relationships between identical or near identical sequences must be inferred from the sequence data and collection date alone. This is particularly relevant in this analysis that has used a single MCC tree for each cluster to run the transmission inference, and where a relatively narrow time interval (3 years) is used. I think it would be useful to see some measure of the genomic diversity in clusters and some discussion on whether this may influence the main results of the accuracy of the transmission inference in simulated and real-world data.

**Other Minor Comments:**

**Introduction**

- I would be worthwhile to make the distinction here between low-incidence and high-incidence setting in the application of this work, although this has been addressed in the discussion. The authors estimate that the sampling density in their real-world data from Botswana is ~ 16%. This value can be higher in low-incidence or well-resourced settings (e.g., DOI 10.1099/mgen.0.000219).

**Methods**

- Could the authors report any studies or empirical data, or explain the rationale, to support their parameter choices for the simulated data, notably the latent (mean 9 months) and infectious period (mean = 3 months).
- Again, choice of prior parameter choices for TransPhylo, particularly the generation time distribution with mean of 1 year, should be justified. Also were these TransPhylo parameter estimates used for both
- Line 223 – Were all sequences with any number of heterozygous sites excluded? This would be very strict as heterozygous sites can occur in non-mixed infections when within-host clonal diversity is present. I would suggest either excluding only sequences with an excess number of heterozygous sites or using an existing method to detect mixed infection (such as doi: 10.1186/s12864-018-4988-z). I I’m not sure it would be worth conducting the full analysis again to include these sequences as it will not likely affect the results of this study though.
- Line 186 – please include the code for this new approach. Given the recommendation to use this

**Results**

- Line 247 – Explain the term ‘frequentist coverage’ and how to interpret the value. I can see this work will be of interest to epidemiologists and biologists with less statistical background so it would be helpful to include a small explanation in the text.
- Line 297 – I would suggest moving this whole section to methods rather than results.

**Supplementary**

- Figure A1 – I would suggest placing the legend for each plot next to plot is relates to, rather than together as it makes it a little hard to read.
